# Exploring cancer knowledge and sources of information among the public: An analytical study

**DOI:** 10.4102/phcfm.v17i1.4820

**Published:** 2025-07-07

**Authors:** Flemmings F. Ngwira, Lusizi Kambalame, Wellman Kondowe, Jessie Mkandawire

**Affiliations:** 1 Department of Language and Communication, School of Education, Communication and Media Studies, Malawi University of Business and Applied Sciences, Blantyre, Malawi; 2 Department of Language, Cultural and Creative Studies, Faculty of Humanities and Social Sciences, Mzuzu University, Mzuzu, Malawi; 3 Department of Public and Environmental Health Sciences, School of Science and Technology, Malawi University of Business and Applied Sciences, Blantyre, Malawi

**Keywords:** cancer, knowledge, communication, risk factors, early warning signs

## Abstract

**Background:**

Malawi faces a heavy cancer burden because of high incidence and late-stage diagnoses, largely driven by low public awareness of cancer risk factors and early warning signs.

**Aim:**

This study aimed to explore the cancer knowledge of cancer risk factors and early warning signs, and sources of information among the public.

**Setting:**

This study was conducted in four districts within the Southern Region of Malawi.

**Methods:**

The study used a cross-sectional approach to elicit knowledge of cancer and sources of cancer information among a sample of 305 participants. Data were collected using a previously standardised Cancer Awareness Measure (CAM). Statistical data analyses were conducted using IBM^®^ SPSS^®^ statistics version 22.

**Results:**

Awareness of cancer risk factors and early warning signs was found to be low, indicating a significant lack of public knowledge about cancer. The radio emerged as the most common medium of cancer information through which Malawians receive cancer information, followed by clinics and hospitals. Interestingly, many individuals perceived clinics – not the radio – as the primary source where they gained a clearer understanding of cancer information.

**Conclusion:**

The study concludes that public knowledge of cancer in Southern Malawi is low and although radio is the main information source, clinics are more effective because of expert guidance.

**Contribution:**

This study identifies critical gaps in cancer awareness and understanding in Malawi, highlighting the need for improved and targeted communication strategies, particularly among vulnerable populations.

## Introduction

Cancer is one of the leading killer diseases in the world and it kills more people in sub-Saharan Africa than HIV/AIDS, tuberculosis and malaria combined.^1^ Malawi is one of the sub-Saharan countries with over 20.9 million population^2^ and it has not been exempted from the cancer epidemic. For instance, Dr Masamba, Head of Oncology at Queen Elizabeth Central Hospital (QECH), lamented that ‘QECH, being a hospital that sees the largest number of at least 200 cancer patients in a day, is facing challenges of limited space to accommodate patients, inadequate staff and shortage of drugs because of the growing number of patients’.^3^ Literature indicates that in many sub-Saharan countries, cancer incidence is very high because most vulnerable populations, such as the uneducated, the elderly and rural residents, are likely to experience barriers to effective health communication such as low literacy levels, limited access and ability to use key communication channels and as a result, they lack proper knowledge of cancer, its early warning signs and risk factors.^4^ Eventually, they become more exposed to avoidable risk factors such as alcohol and tobacco use^5^ and usually report late for medical treatment.^6,7^

Knowledge of cancer risk factors is an important aspect in developing cancer risk perceptions and influencing the participation of health protection behaviours such as cancer screening, avoiding smoking and following a low-fat, high-fibre diet.^8^ Cancer screening helps health professionals detect and treat cancer timely. Besides cancer screening, knowledge about early warning signs is vital for the early diagnosis of cancer. According to Kreps,^9^ studies have shown that knowledge of early warning signs of cancer could lead to timely presentation of the illness to the hospital, which could eventually result in a timely diagnosis and treatment. It is of particular importance for people to have proper knowledge of early warning signs, especially in Malawi where because of economic constraints, cancer screening is limited to only breast and cervical cancers.^10,11^ Conversely, a lack of cancer awareness may fuel myths and misconceptions, which may influence the way people perceive cancer and eventually this perception affects disease control. Therefore, we could postulate that one of the major factors contributing to such a high cancer incidence and late presentation in sub-Saharan Africa and Malawi in particular, might be lack of awareness of risk factors and early warning signs.

For people to be aware of cancer and its risk factors, they need to be exposed to relevant information that can influence them to take action towards their health protection. Health information does not always need to be generated and exchanged in clinics and hospitals. Other channels such as the radio, TV and schools are equally vital for health education and promotion. Research shows that mass media plays an essential role in influencing the public’s awareness and perception of cancer.^12^ Furthermore, as Kreps^4^ argues, health communication settings should also include places such as homes, schools, offices and any other place people gather for a common goal. Research reveals that local leaders have high credibility and are often ideal sources of cancer prevention messages.^13,14^ To be effective at disseminating cancer prevention messages, it is important to increase the amount of available information on the topic of interest by delivering messages at several points in time.^15^ Through this strategy, which Kreps^9^ refers to as ‘multiple reinforcing messages’, information is delivered via different complementary channels at various times, with each message reinforcing key ideas through repetition and alignment.

To our knowledge, studies addressing the awareness of cancer risk factors, early warning signs and sources of information in Malawi appear to be rare. Most previous relevant studies concentrated on either cancer awareness,^6^ help-seeking behaviours, especially for cancer screening services,^10,11^ incidence^16,17^ or treatment.^18^ Apart from a few such studies concerning awareness, cancer screening, incidence and treatment, very little or nothing is known about the public’s cancer knowledge and their sources of cancer information in Malawi. According to the Integrative Model (IM) of behaviour prediction, which is a behavioural change model, any given behaviour is most likely to occur if one has a strong intention to perform it.^19^ This implies that the desired behaviour may not be performed when people are not aware of the reasons for performing the behaviour and have not yet formed the intentions to perform it. Guided by the theory, and stimulated by the neglect of research into awareness of cancer and sources of information, this study was set to investigate knowledge of cancer risk factors and early warning signs, and sources of cancer information among people with various socioeconomic statuses and diverse backgrounds in the southern region of Malawi; the country has three main regions, northern, central and southern.

## Research methods and design

### Study design, aim and objectives

The study employed a quantitative cross-sectional approach to assess public knowledge of cancer risk factors and early warning signs, as well as the sources of cancer information in Malawi.

### Setting

The study was conducted in the southern region of Malawi and the four chosen districts were Blantyre, a commercial city and three other rural districts, namely, Chikwawa, Thyolo and Chiradzulu. The southern region was particularly selected for this research because it registered the highest cancer incidence in a nationwide cancer registry.^17^

### Study population and sampling strategy

The study population consisted of all men and women, aged 15 and older, not diagnosed with any type of cancer. A systematic random sampling approach was used to identify a heterogeneous sample of 310 participants (154 from Blantyre and 156 participants from the three rural districts – approximately 52 participants from each district) to participate in the survey.

### Data collection

For data collection, previously standardised and published Cancer Awareness Measure (CAM) with an overall internal reliability of Cronbach’s alpha 0.77^20^ was adapted to investigate cancer’s common early warning signs and risk factors, and sources of cancer information. The original instrument comprises nine main questions with a total of 47 open and closed items, but for this research, only 4 questions with 22 items addressing the study constructs of cancer risk factors and early warning signs were used. To investigate people’s sources of information, three questions with 10 items were developed based on the literature and were added to the CAM instrument. In total, the newly adapted CAM for the research comprised 7 questions with 32 items. Before conducting the actual study, a pilot study was carried out to test the research instruments and ensure their reliability and effectiveness in assessing cancer awareness in Malawi. Finally, the survey was conducted in the local language, Chichewa, with the questionnaire being translated and back-translated for accuracy. Participants took approximately 11 min to complete the assessment.

### Data analysis

Statistical data analyses were conducted using IBM^®^ SPSS^®^ statistics version 22. Before the data analysis, the collected data were screened for accuracy and missing values. To ensure the instruments’ internal consistency on the current sample, reliability analysis using Cronbach’s alpha was calculated and reported. Pearson correlations were calculated to establish the association between variables assessed in the study. Descriptive statistics for all the tested variables were realised and tabulated from raw data. To test if there were disparities in the studied variables between and among population sub-groups, independent samples *t*-test, Chi-square and the Analysis of Variance (ANOVA) were used (*p* < 0.05).

### Ethical considerations

Before the survey was conducted, the research protocol adhered to key ethical standards aimed at safeguarding participants’ lives, privacy, and confidentiality. Ethical clearance to conduct this study was obtained from the Ministry of Health and Population, National Health Sciences Research Committee (NHSRC) (No. 1881). Authorisation to carry out the study in Blantyre, Thyolo, Chikwawa, and Chiradzulu districts was granted by the respective District Commissioners and Traditional Chiefs. Verbal and written informed consent were secured from all participants. To ensure anonymity, no identifying personal information was collected during the study. Due to the limited cancer-related vocabulary in the local language and the participants’ likely unfamiliarity with cancer terms, trained interviewers, guided by Oncology professionals, administered the questionnaire and helped clarify complex terms and expressions.

## Results

Out of a sample of 310, only 5 respondents (1.6%) indicated that they had never ever heard about cancer and were excluded from the survey. A total number of 305 southern region residents participated in this study and they all completed the survey. There was a good balance between male (*n* = 143) and female (*n* = 162) participants, and those from urban (*n* = 154) and rural (*n* = 151) areas. Regarding the age-range, the majority were young participants (15–30 years *n* = 137; 31–40 years *n* = 97; 41–50 *n* = 42; 51 and above *n* = 29). Concerning marital status, the majority were married (*n* = 169), seconded by those who were single (*n* = 87), followed by divorced (*n* = 28) and widowed (*n* = 21). On education status, the majority were those with secondary education (*n* = 116), then tertiary (87), primary (*n* = 81) and none (*n* = 21). Regarding employment status, most participants were self-employed (*n* = 110), followed by unemployed (*n* = 84), employed (*n* = 73) and students (*n* = 38). The CAM demonstrated good internal reliability, with a Cronbach’s alpha of 0.72.

### Knowledge of cancer’s early warning signs and risk factors

Responses to the open (recall) and closed (recognition) questions about early warning signs and risk factors are shown separately in Table 1 and Table 2. In the recall (unprompted) format, where respondents were asked to recall their knowledge of cancer, few respondents were able to mention common early warning signs and risk factors of cancer. For instance, some items were mentioned by far fewer respondents as in the case of ‘persistent change in bowel or bladder habits’ (0.7%), and others, no respondent mentioned as in the case of ‘change in mole appearance’ (0.0%). In the recognition (prompted) format, where respondents were asked to recognise a given common early warning sign and risk factor of cancer, respondents’ recognition was higher than recall, as expected.

**TABLE 1:** Awareness of cancer by recall and recognition of its early warning signs (*N* = 305).

Early warning signs	Recall (unprompted)		Recognition (prompted)	
	Frequency	%	Frequency	%
Sore that does not heal	137	44.9	274	89.8
Unexplained lump or swelling	87	28.5	207	67.9
Persistent unexplained pain	47	15.4	86	28.2
Unexplained bleeding	32	10.5	194	63.6
Unexplained weight loss	12	3.9	70	23.0
Persistent difficult swallowing	11	3.2	174	57.0
Persistent cough or hoarseness	9	3.0	98	32.1
Persistent change in bowel or bladder habits	2	0.7	109	35.1
Change in mole appearance	0	0.0	167	54.8

**TABLE 2:** Awareness of cancer by recall and recognition of risk factors (*N* = 305).

Risk factors	Recall (unprompted)		Recognition (prompted)	
	Frequency	%	Frequency	%
Smoking any cigarette	82	26.9	271	88.9
Unprotected sex (infection with HPV)	69	22.6	193	63.3
Excessive alcohol intake	36	11.8	177	58.0
Exposure to intense sun rays	15	4.9	135	44.3
Doing less physical activities	3	1.0	122	40.0
Exposure to someone’s smoke	2	0.7	202	66.2
Eating less fruits and vegetables	2	0.7	124	40.7
Eating more red or processed meat	2	0.7	142	46.6
Being over 70 years of age	2	0.7	75	24.6
Having a close relative with cancer	2	0.7	71	23.3
Being overweight	1	0.3	96	31.5

HPV, Human Papillomavirus.

To test if there were significant differences between the total mean number of early warning signs and risk factors on recall and recognition, one-way repeated measures of ANOVA were used. Using the uncorrected results denoted as ‘Sphericity Assumed’, the *F*-test results indicate that early warning signs and risk factors were not recalled equally (*F*[1304] = 40.65, *p* < 0.001), which indicates that the total mean number of early warning signs (*M* = 1.10) was significantly higher than risk factors (*M* = 0.71). The *F*-test results also show that early warning signs and risk factors were not recognised equally (*F*[1304] = 28.83, *p* < 0.001); the total mean number of risk factors (*M* = 5.27) was significantly higher than early warning signs (*M* = 4.52).

### Demographic differences in cancer knowledge

Chi-square (χ^2^) tests were used to examine differences between and among groups for each early warning sign and risk factor of cancer. Because of the failure to ‘recall’ early warning signs and risk factors by the majority of the participants, χ^2^ tests could not be performed on cells, which had an expected count of less than five numerous items. Consequently, χ^2^ tests were performed only on prompted responses, which involved participants’ recognition of the early warning signs and risk factors. A general analysis of Chi-square results for significant differences between and among groups in prompted (recognition) responses reveal that there were very few significant differences among demographic groups in recognising early warning signs and risk factors. For instance, out of all the nine early warning signs and 11 risk factors, only one early warning sign, ‘lump or swelling’ showed that there were significant differences among respondents of different ages (χ^2^ [3305] = 13.64, *p* < 0.01) with ages of 41–50 (85.7%) recalling it the most.

To assess how different sub-groups recalled and recognised early warning signs and risk factors of cancer in total, a one-way ANOVA was used. Analysis of Variance results regarding recall of early warning signs revealed that there were significant differences within categories of residence (*F*[1303] = 8.79, *p* < 0.01) and education (*F*[3301] = 5.13, *p* < 0.01). Urban residents (*M* = 1.25) and participants with tertiary education (*M* = 1.38) recalled more early warning signs than their rural residents (*M* = 0.95) and those with either no or low formal education (none [*M* = 0.67]; primary [*M* = 1.00]; secondary [*M* = 1.05]), respectively. Similarly, concerning the recall of risk factors, there were significant differences within categories of gender (*F*[1303] = 4.15, *p* < 0.05), residence (*F*[1303] = 6.70, *p* < 0.01) and education (*F*[3301] = 10.95, *p* < 0.001). Male participants (*M* = 0.82), urban residents (*M* = 0.84) and participants with tertiary education (*M* = 1.09) mentioned more risk factors than female participants (*M* = 0.61), rural residents (*M* = 0.58) and those with either no or low formal education (none [*M* = 0.33]; primary [*M* = 0.40]; secondary [*M* = 0.71]), respectively. Table 3 and Table 4 show ANOVA results in detail, focusing on demographic differences in cancer awareness by recall and recognition of its early warning signs and risk factors.

**TABLE 3:** Analysis of Variance results showing demographic differences of identified early warning signs (*N* = 305).

Groups	Number of early warning signs recalled					Number of early warning signs recognised				
	Estimated marginal mean	95% CI	Significance			Estimated marginal mean	95% CI	Significance		
			*F*(1303)	*F*(3301)	*p*-value			*F*(1303)	*F*(3301)	*p*-value
**Gender**	-	-	0.41	-	0.51	-	-	4.51	-	0.05
Male	1.07	0.92–1.21	-	-	-	4.79	4.43–5.14	-	-	-
Female	1.13	0.99–1.27	-	-	-	4.28	3.97–4.59	-	-	-
**Residence**	-	-	8.79	-	0.01	-	-	0.41	-	0.43
Urban	1.25	1.09–1.40	-	-	-	4.42	4.10–4.75	-	-	-
Rural	0.95	0.82–108	-	-	-	4.61	4.27–4.95	-	-	-
**Age (years)**	-	-	-	0.76	0.52	-	-	-	0.71	0.54
15–30	1.04	0.88–1.20	-	-	-	4.34	3.98–4.69	-	-	-
31–40	1.09	0.91–1.27	-	-	-	4.65	4.22–5.08	-	-	-
41–50	1.24	0.99–1.48	-	-	-	4.79	4.17–5.40	-	-	-
> 51	1.24	0.91–1.57	-	-	-	4.59	3.81–5.36	-	-	-
**Education**	-	-	-	5.13	0.01	-	-	-	1.64	0.18
None	0.67	0.37–0.97	-	-	-	3.62	2.67–4.57	-	-	-
Primary	1.00	0.82–1.19	-	-	-	4.74	4.22–5.27	-	-	-
Secondary	1.05	0.90–1.20	-	-	-	4.49	4.16–482	-	-	-
Tertiary	1.38	1.17–1.59	-	-	-	4.57	4.12–5.02	-	-	-

Cl, confidence interval.

**TABLE 4:** Analysis of Variance Analysis of Variance results showing demographic differences of identified risk factors (*N* = 305).

Groups	Number of risk factors recalled					Number of risk factors recognised				
	Estimated marginal mean	95% CI	Significance			Estimated marginal mean	95% CI	Significance		
			*F*(1303)	*F*(3301)	*p*-value			*F*(1303)	*F*(3301)	*p*-value
**Gender**	-	-	4.15	-	0.05	-	-	9.38	-	0.01
Male	0.82	0.67–0.97	-	-	-	5.72	5.32–6.11	-	-	-
Female	0.61	0.48–1.74	-	-	-	4.87	4.50–5.25	-	-	-
**Residence**	-	-	6.70	-	0.01	-	-	0.41	-	0.19
Urban	0.84	0.69–0.99	-	-	-	5.09	4.73–5.45	-	-	-
Rural	0.58	0.44–0.71	-	-	-	5.46	5.04–5.87	-	-	-
**Age (years)**	-	-	-	1.96	0.12	-	-	-	0.88	0.45
15–30	0.77	0.62–0.93	-	-	-	5.04	4.63–5.45	-	-	-
31–40	0.66	0.48–0.84	-	-	-	5.46	4.95–5.98	-	-	-
41–50	0.83	0.54–1.12	-	-	-	5.33	4.72–5.94	-	-	-
> 51	0.38	0.61–0.81	-	-	-	5.66	4.66–6.65	-	-	-
**Education**	-	-	-	10.95	0.001	-	-	-	0.74	0.53
None	0.33	0.62–0.93	-	-	-	4.95	3.65–6.26	-	-	-
Primary	0.40	0.48–0.84	-	-	-	5.60	4.99–6.22	-	-	-
Secondary	0.71	0.54–1.12	-	-	-	5.15	4.72–5.59	-	-	-
Tertiary	1.09	0.61–0.81	-	-	-	5.20	4.76–5.63	-	-	-

Cl, confidence interval.

During closed-ended questions, respondents were asked to endorse where and how often they get cancer information, and the responses are shown in Table 5. The most commonly endorsed source of cancer information where respondents ‘oftentimes’ get cancer information was the radio (57.7%) seconded by clinics, hospitals or dispensaries (operationally defined here as ‘the clinic’; 51.5%). The least-endorsed source of information was religious gathering (5.2%). Chi-square results, especially on the commonly accessed sources of cancer information, namely the radio and the clinic, revealed that there were no significant differences between demographic subgroups with regard to the radio. However, a significant difference was noticed regarding the clinic, χ^2^ (1305) = 5.35, *p* = 0.05, with 56.2% females accessing it against 46.2% males.

**TABLE 5:** Cancer information sources in the past year (*N* = 305).

Source of information	Never		Sometimes		Oftentimes	
	*n*	%	*n*	%	*n*	%
Radio	28	9.2	101	33.1	176	57.7
Television	115	37.7	101	33.1	89	29.2
Newspapers or magazines	113	37.0	122	40.0	70	23.0
Clinic, hospital or dispensary	33	10.8	115	37.7	157	51.5
School, work or business place	128	42.0	136	44.6	41	13.4
Religious gathering (church or mosque)	174	57.0	115	37.7	16	5.2
Community gatherings	150	49.2	127	41.6	28	9.2
Family members and friends	106	34.8	159	52.1	40	13.1

Using an open-ended question, respondents were also asked to indicate the source from which they thought they understood most of the information, and provide the reasons. The most-endorsed source was the clinic with 53.8%, followed by the radio with 25.9%. Other sources, such as family and friends, and religious gatherings were the least mentioned with lower endorsement of 2.3% and 1.3%, respectively. Regardless of the source of information, the most cited reasons, which aided their better understanding were ‘clear explanations’ (32.1%) and ‘health experts speaking’ (25.2%). Other reasons such as ‘availability of the medium’ (17%), ‘freedom to ask questions’ (10.2%) and ‘availability of illustrations and testimonies’ (7.5%) were also mentioned, but by a few participants. Cross-tabulation results showed that for the clinic alone, the most frequent reasons participants cited were ‘health experts speaking’ (40.9%) and ‘clear explanations’ (29.9%); and for the radio alone, the most frequent reasons respondents mentioned were ‘availability of the medium’ (39.2%). Especially on the radio, the majority (71%) of participants described the messages as very brief and monotonous; the radio keeps on broadcasting the same messages with little substance in it.

## Discussion

In cancer care, health communication is essential for prevention, early detection, psychological support, treatment guidance and end-of-life care. This study investigated the awareness of cancer risk factors and early warning signs, and sources of information among the general public in Malawi. Results of the study revealed that there is low awareness of risk factors and early warning signs. Especially with the open-ended questions (recall format), scores were relatively poor for all risk factors (0.3% < 27%) and all early warning signs (0.00% < 29%) except for ‘a sore that does not heal’, which was recalled by only 44.9% of the respondents. However, recognition scores of risk factors and early warning signs were higher with ‘smoking any cigarette’ (a risk factor) and ‘a sore that does not heal’ (an early warning sign) being identified by 88.9% and 89.8% of the respondents, respectively. Interestingly, even in the recognition format, most respondents were able to recognise only three to four early warning signs or risk factors. This study postulates a strong indication that there is limited knowledge of cancer among the general public in Malawi. These results have significant implications given the high incidence of cancer in the country.

Concerning the limited knowledge, our findings support previous related studies conducted in Malawi,^21,22^ Africa^23,24^ and the developed countries.^25,26^ This indicates that awareness of cancer continues to be low in many parts of the world and is worse in Africa. Studies in Malawi,^17,21,22^ for instance, show that public awareness has remained low since 2012, with little progress made to improve the situation. According to the IM theory,^19^ a lack of cancer awareness would lead to lack of intention towards help-seeking behaviours among individuals. Furthermore, Fishbein and Yzer^19^ argue that the most important aspect in predicting attitudes, intentions and behaviours is the accessibility of beliefs in one’s memory. This assumption would suggest that early warning signs and risk factors that were recalled in response to the survey’s open-ended questions are more likely to predict health protective behaviours such as help-seeking than those that were just recognised. Lessons can be drawn from the effectiveness of past health promotion strategies in Malawi, as noticed by Ngwira et al.^27^ For instance, during the Breast Cancer Awareness Week in 2019, awareness significantly increased because of the dominance of breast cancer messages in mass media coverage. This suggests that a multi-channel approach increases cancer public awareness.

Differences between some demographic groups were noticed, highlighting particular gaps in awareness. According to the IM theory, these demographic factors affect cancer awareness and influence behaviour. The findings suggest that cognitive models of cancer, and especially the understanding of early warning signs and risk factors, may vary between groups, possibly because of the different demographic attributes that exist among groups. However, it should also be observed that these differences between groups do overlap because, for instance, one participant might be male, educated and may live in an urban area. Regarding education status, consistent with other studies,^28,29^ the study reveals that education has a significant association with recall knowledge of early warning signs and risk factors; the knowledge increases with increasing formal education levels. Education improves understanding of health issues; it is linked with people’s self-perceptions of the disease and the understanding of health education and counselling that goes on with it. As Peterson et al.^30^ argue, low literacy levels are associated with less knowledge about cancer; illiteracy hinders individuals from understanding health-promotion messages, particularly those who require reading and interpreting complex information.

Place of residence and gender were also noticed to influence participants’ cancer knowledge. Regarding place of residence, poorer knowledge was observed for participants living in rural areas. Urban residents might have taken advantage of the wide variety of communication media, such as newspapers and TV, to advance their cancer knowledge. People in rural settings are what Kreps^4,9^ refers to as the most vulnerable population who need to be reached with messages of cancer prevention and control. Most vulnerable populations in Malawi face significant health communication barriers, including low health literacy, limited access to information, and difficulty using digital communication channels, which can hinder their access to cancer prevention messages. Concerning gender, poorer knowledge was observed among female participants. The finding is different from what was obtained in the UK population-based survey.^25^ These gender differences could be attributed to different levels of education between male and female citizens. Gender gaps in education still exist in Malawi despite current efforts to have equal access to education. Girls are twice as likely as boys to drop out of school because of several sociocultural and economic factors, such as poverty and early pregnancies, that affect them more than boys.^31^

Concerning cancer information sources, findings indicate that the radio is the most common source of cancer information for most Malawians, followed by the clinic. This trend is consistent across both rural and urban areas, highlighting the radio’s widespread reach because of its portability, simplicity and low cost, especially in regions with unreliable electricity. Several studies^15,32,33^ have documented the role mass media, especially the radio, plays in promoting health. Although people indicated it as the second common source, the clinic also plays a crucial role, particularly for women, who frequent it more often because of reproductive health needs.^28,34^ Health advocates should leverage these primary sources to enhance public knowledge.

Interestingly, while the radio is widely used, people find the clinic to be more effective for understanding health information because of the presence of health experts and clear explanations. Research^28,34,35^ reveals that people often trust health information when it comes from the experts themselves, like doctors and nurses. Despite the radio’s extensive use, its brief and repetitive messages often fail to satisfy listeners’ need for comprehensive information. Effective communication should go beyond mere repetition to enhance knowledge and promote behavioural change. When used effectively, the radio could enhance knowledge, attitude, beliefs and awareness, leading to behavioural change.^36,37^ Moreover, interpersonal communication channels such as schools, workplaces and community gatherings are underutilised, leading to missed opportunities for disseminating cancer information. Kreps^4^ argues that health-promotion messages should go as far as schools and workplaces; these are places where children (in schools) and adults (at workplaces) spend much of their time. Moreover, taking advantage of the interpersonal communication humanistic effect on an individual, it is important to increase the amount of available information by delivering messages using a variety of communication channels.

Findings from this research provide empirical evidence on the centrality of health communication in cancer prevention and highlight the need for strategic awareness communication, which, according to Kreps,^4,9^ entails reaching and influencing the target audience, which is, in most cases, the vulnerable population. For strategic communication to be successful, research posits that it is essential to begin with a careful needs and audience analysis to identify the best goals, targets and strategies for communication interventions.^9^ Without this information about the target population, it is certainly difficult to develop appropriate cancer awareness messages that are effective and influential. The need for strategic communication about cancer prevention in Malawi is particularly critical and yet challenging. With the limited cancer knowledge among the population, coupled with low education levels and limited resources among most people, communication about cancer prevention is bound to face several challenges. To curb the adverse situation, Kreps^9^ postulates that communicators need to develop clear and easy-to-understand communication strategies. Helping healthcare consumers navigate the complexity of cancer can enhance their understanding of its various types, causes, stages, early warning signs and risk factors, empowering them to make informed health decisions and adopt protective behaviours.

### Limitations

There are a few constraints of this study and conclusions drawn from this analysis must be interpreted with some considerations of the highlighted limitations. To begin with, the findings of this study are based on survey interviews from four districts of Blantyre, Chikwawa, Thyolo and Chiradzulu, all located in the Southern Region of the country. Because of the large target study population for the study (the public), the sample recruited in this study, therefore, may not adequately represent the larger population (nationwide). The country has diverse demographic factors, such as culture among different regions. Therefore, the interpretation of the findings from the study may be limited to the southern region from which data were gathered. The research process and design used for the study, however, may still provide a framework for understanding, exploring, and fostering cancer communication in other contexts. Future comprehensive studies need to concentrate on a nationwide sampling that may be fully representative of the entire country on the general public’s level of cancer knowledge and where they get cancer information.

Furthermore, this was a cross-sectional study, where data were collected and analysed at one point in time. This means that because of its cross-sectional nature, the study did not investigate whether cancer awareness and health behaviours change over time. As individuals are dynamic, their attitudes, beliefs and behaviours towards their health and well-being may change over time. Future studies should aim at investigating the stability of the general public regarding what they know about cancer and their sources of information. Finally, another key limitation of this study is that it focused solely on measuring cancer awareness using the CAM without assessing attitudes and behaviours related to cancer prevention and early detection (CAM-Plus). As a result, the findings provide insights into knowledge levels but do not capture whether awareness translates into proactive health-seeking behaviours or changes in perception. Future research should incorporate CAM-Plus to explore the relationship between awareness, attitudes and behaviours, providing a more comprehensive understanding of cancer-prevention efforts.

### Recommendations

To address the low public awareness of cancer risk factors and early warning signs in Malawi, targeted interventions should be implemented through awareness campaigns, community engagement and policy enhancements. Awareness campaigns should leverage radio as the most widely accessed information source, and social media and mobile-based messaging (for the younger demographics), incorporating interactive discussions, expert interviews and survivor testimonies to enhance public understanding. Community engagement should be strengthened through health outreach programmes using clinics, hospitals, community health workers, schools, religious institutions and local leaders, to provide face-to-face education to dispel myths and encourage early screening. At the policy level, cancer awareness should be integrated into national health education programmes, and healthcare workers should receive training in effective cancer communication. In addition, the government should establish community-based cancer screening initiatives and strengthen partnerships with Non-governmental organisations (NGOs) and international health agencies to secure sustainable funding for long-term awareness efforts. By implementing these recommendations, Malawi can improve public knowledge of cancer, promote early detection, and ultimately reduce its cancer burden.

## Conclusion

This study offers significant insights for culturally relevant health promotion in Malawi, particularly regarding cancer awareness. The findings emphasise a critical need for clear, accessible cancer information to encourage informed health decisions and address widespread misconceptions. Public health professionals are responsible for providing comprehensive yet simplified information on cancer risk factors, symptoms and preventive measures, helping to demystify and destigmatise the topic, which is often associated with fear and confusion. Effective communication must consider the emotional impact of cancer information and aim to avoid causing fear or alienation among the public, particularly among vulnerable populations.

The study also highlights the role of radio and clinics in delivering cancer messages. With its broad reach, the radio should convey persuasive messages that motivate protective health behaviours, as current brief, repetitive messages lack impact. Meanwhile, clinics are identified as key sources of comprehensible information, where the presence of health professionals fosters trust and understanding. This underscores the value of healthcare professionals in health promotion, suggesting that both mass media and interpersonal channels should include expert involvement. Interactive programmes, such as radio call-ins, could further bridge the gap between providers and the public, enhancing message effectiveness and fostering consumer confidence in cancer prevention.
